# Supplementary material for: An engineered disulfide staple restricts lid loop dynamics and alters substrate specificity of phenylalanine ammonia-lyase
Source: bioRxiv. 2026 May 6:2026.05.01.722275. Preprint. [Version 1] doi: 10.64898/2026.05.01.722275 (PMC13174424; doi:10.64898/2026.05.01.722275)
Supplement: Supplement 1 [file media-1.pdf]

Supplemental Information

An engineered disulfide staple restricts lid loop dynamics and alters substrate specificity of phenylalanine ammonia-lyase

Rebecca Condruti[1], Likith Muthuraj[2], Jeevan K. Prakash[2], Samuel D. Littman[3], Pravin Kumar R.[2*], Nikhil U. Nair[1,4,*]

1. Graduate School of Biomedical Sciences, Tufts University, Boston, MA 02111, United States
2. Department of Protein Design and AI, Kcat Enzymatic Private Limited, Bengaluru 560005, Karnataka, India
3. Department of Chemistry, Tufts University, Medford, MA 02155, United States
4. Department of Chemical & Biological Engineering, Tufts University, Medford, MA 02155, United States

[*] Correspondence: pravin.k@kcat.co.in; nikhil.nair@tufts.edu

RUNNING TITLE

Disulfide Staple Alters Substrate Specificity of PAL

Figure List:

1. Supplemental Figure 1: Principal Component Analysis (PCA) of enzyme dynamics.
2. Supplemental Figure 2: Description of expression conditions for *Av*PAL.

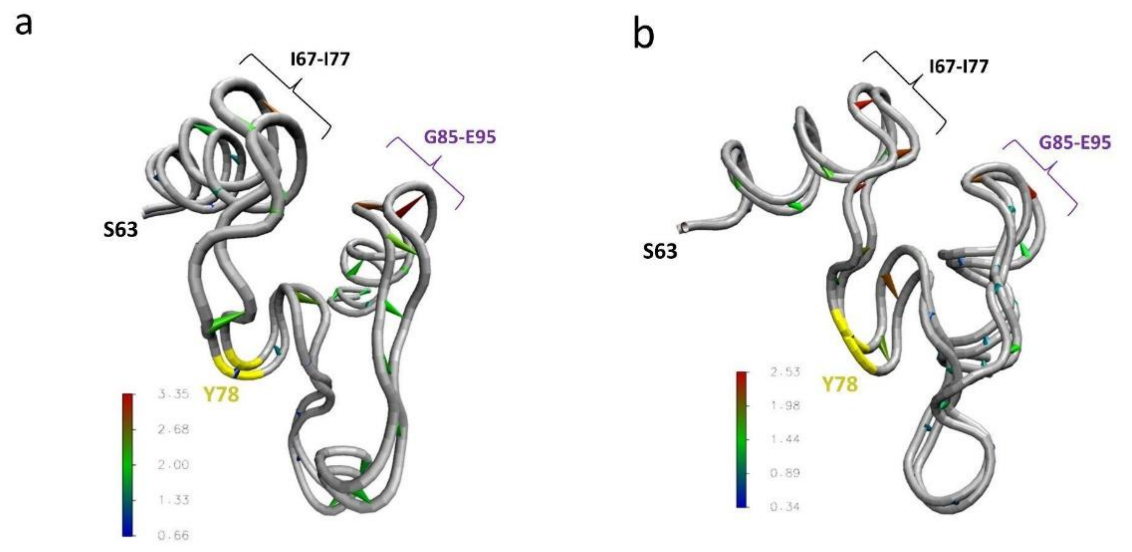

Figure S1. Principal Component Analysis (PCA) of enzyme dynamics.
(a) Porcupine plot for the WT enzyme from metadynamics, showing significant motion in the inner-lid loop. (b) Porcupine plot for L4 from metadynamics, illustrating a more constrained motion profile with reduced high-amplitude fluctuations, indicating increased rigidity in the inner-lid loop. Red indicates the highest movement, followed by green (moderate) and blue (least). Arrows represent the direction of correlated motion.

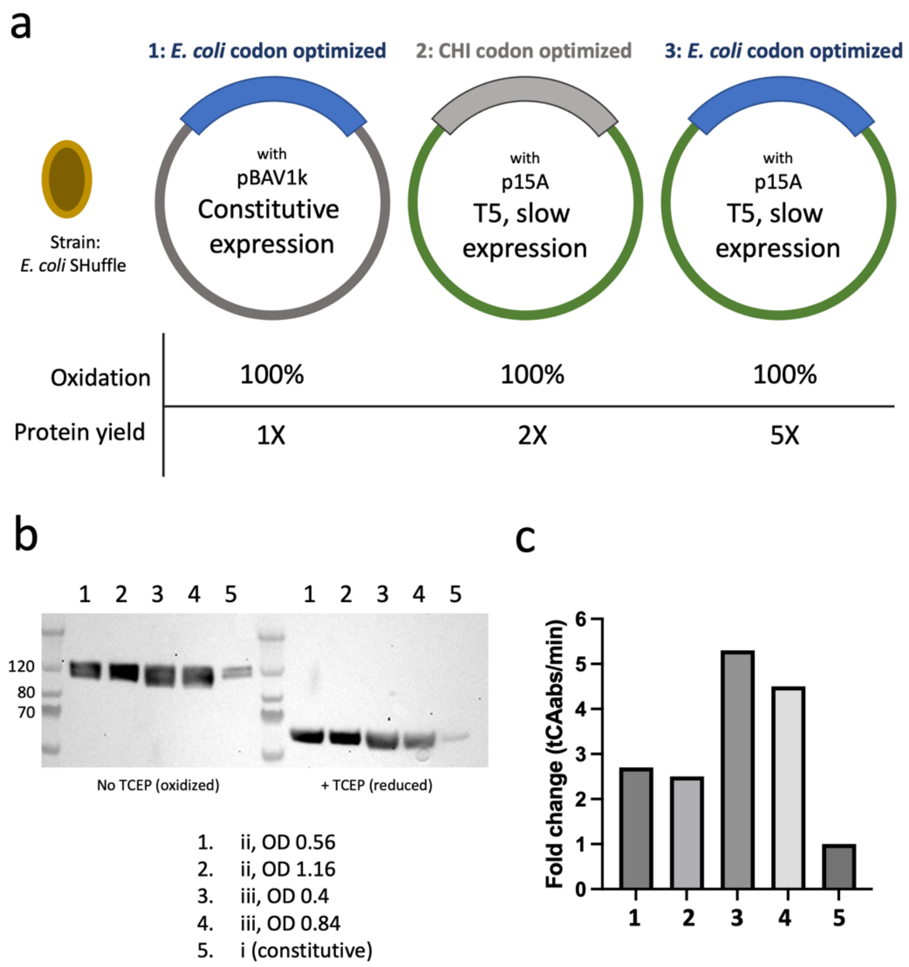

Figure S2. Description of expression conditions for *Av*PAL.
(a) Cartoon representation of paired codon optimization strategy with vector expression (1). Protein yield was best with basic codon optimization and T5 inducible expression as compared to previously published methods (2). (b) Western blot of protein yield from lysate, normalized to total lysate concentration. (c) Fold change of enzyme activity from lysate, normalized to total lysate.

References

1. Love, A. M., and Nair, N. U. (2024) Specific codons control cellular resources and fitness. *Science Advances*. **10**, eadk3485
2. Mays, Z. J., Mohan, K., Trivedi, V. D., Chappell, T. C., and Nair, N. U. (2020) Directed evolution of Anabaena variabilis phenylalanine ammonia-lyase (PAL) identifies mutants with enhanced activities. *Chem Commun (Camb)*. **56**, 5255–5258
